# Prediction of protein interactions with function in protein (de-)phosphorylation

**DOI:** 10.1371/journal.pone.0319084

**Published:** 2025-03-03

**Authors:** Aimilia-Christina Vagiona, Sofia Notopoulou, Zbyněk Zdráhal, Mariane Gonçalves-Kulik, Spyros Petrakis, Miguel A. Andrade-Navarro

**Affiliations:** 1 Faculty of Biology, Insitute of Organismic and Molecular Evolution, Johannes Gutenberg University, Biozentrum I, Mainz, Germany; 2 Institute of Applied Biosciences/Centre for Research and Technology Hellas, Thessaloniki, Greece; 3 Central European Institute of Technology, Masaryk University, Brno, Czech Republic; UC Los Angeles: University of California Los Angeles, UNITED STATES OF AMERICA

## Abstract

Protein–protein interactions (PPIs) form a complex network called “interactome” that regulates many functions in the cell. In recent years, there is an increasing accumulation of evidence supporting the existence of a hyperbolic geometry underlying the network representation of complex systems such as the interactome. In particular, it has been shown that the embedding of the human Protein-Interaction Network (hPIN) in hyperbolic space (H^2^) captures biologically relevant information. Here we explore whether this mapping contains information that would allow us to predict the function of PPIs, more specifically interactions related to post-translational modification (PTM). We used a random forest algorithm to predict PTM-related directed PPIs, concretely, protein phosphorylation and dephosphorylation, based on hyperbolic properties and centrality measures of the hPIN mapped in H^2^. To evaluate the efficacy of our algorithm, we predicted PTM-related PPIs of ataxin-1, a protein which is responsible for Spinocerebellar Ataxia type 1 (SCA1). Proteomics analysis in a cellular model revealed that several of the predicted PTM-PPIs were indeed dysregulated in a SCA1-related disease network. A compact cluster composed of ataxin-1, its dysregulated PTM-PPIs and their common upstream regulators may represent critical interactions for disease pathology. Thus, our algorithm may infer phosphorylation activity on proteins through directed PPIs.

## Introduction

Protein–protein interactions (PPIs) play crucial roles in fundamental processes in living cells [1,2]. PPIs in cells form a complicated network which has been named “interactome” [3]. By coordinating the activity of many proteins and protein complexes, the interactome performs many functions, including signal transduction, cell growth and differentiation, catalytic metabolic reactions, activation or suppression of a protein, transportation of molecules, etc [4,5]. Studying PPIs can help to reveal the underlying molecular machinery in cells [6]. Aberrant PPIs are associated with a wide range of human diseases, including cancer, infectious diseases and neurodegenerative diseases [7,8]. Recent studies indicate that targeting and restoring dysregulated PPIs is a promising strategy for drug development for therapeutic intervention [9,10].

Several studies support that complex networks, such as the interactome, are well suited to be modeled using hyperbolic geometry, a space whose mathematical properties naturally lead to the emergence of networks with scale invariance and strong clustering [11–13]. The Popularity-Similarity (PS) model provides a geometric interpretation in hyperbolic space (H^2^) and assumes that the clustering and hierarchy of complex networks arise from tradeoffs between popularity and similarity of nodes [14]. Basically, in the PS model, the network nodes are situated within a circle at polar coordinates. The network nodes have a radial coordinate that represents their popularity or seniority, the angular coordinate reflects the similarity between nodes, and the hyperbolic distance between nodes abstracts an optimization process in which new nodes connect to nodes that are popular and similar.

Alanis-Lobato et al. found that the embedding of the human Protein-Interaction Network (hPIN) in hyperbolic space has biological interpretations in terms of the PS model. The radial positioning of the nodes encapsulates information about the conservation and the evolution of proteins, corresponding to popularity and seniority, where nodes closed to the center of the circle represent proteins that evolved earlier and had more time to receive connections from newer proteins situated in the periphery of the circle. The angular positioning reflects the functional similarity between proteins and is driven by interactions in pathways and protein complexes, thus capturing the functional and spatial organization in the cell [15]. This mapping can also lead to a better understanding of complex human disorders [16,17].

Information on protein interactions can be obtained by a variety of experimental methods and this data is systematically stored in specialized databases [18–21]. However, little is known about the function of many of these interactions, especially those obtained by high-throughput methods, like yeast-two-hybrid. Following our findings about the biological properties encapsulated in the mapping of the hPIN in hyperbolic space, here we explore if this mapping also contains information that would allow us to predict the function of PPIs.

**Fig 1:**
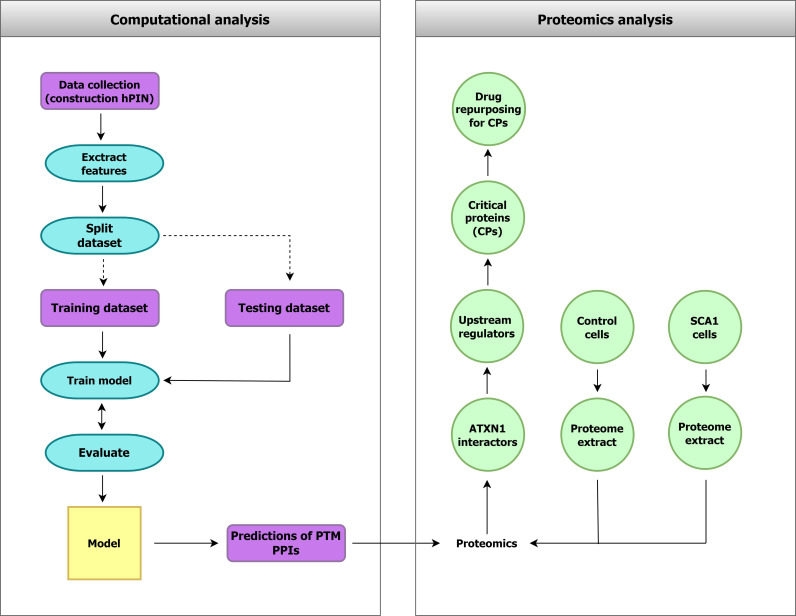
Structure of the work presented in this manuscript. **Left:** computational prediction of PTM-related directed protein interactions (PTM-PPIs). A random forest model was constructed to predict PTM-PPIs based on hyperbolic topological features and network properties extracted from the hPIN. **Right:** quantitative proteomics data revealed dysregulated proteins in a cellular model of SCA1. The results were interpreted using predicted PTM-PPIs, providing directions for future experimental research on SCA1 pathogenesis.

PPIs may result in the post-translational modification (PTM) of one of the interacting proteins. PTMs are considered as covalent or enzymatic modifications of a protein occurring after protein synthesis. They are classified into different groups such as the addition of functional groups/chemical groups (acetylation, methylation, phosphorylation), the addition of a polypeptide chain (ubiquitination, SUMOylation), the addition of other complex molecules (palmitoylation, glycosylation), and amino acid modifications (proteolytic cleavage) [22].

In this work, we applied a machine learning method (random forest, RF) to predict whether PPIs result in PTMs using properties extracted from the mapping of the hPIN in hyperbolic space (Fig 1). To validate the potency of our algorithm, we predicted PTM-related protein interactions (PTM-PPIs) of ataxin-1, a protein implicated in Spinocerebellar ataxia type 1 (SCA1). SCA1 is a severe neurodegenerative disease caused by CAG-trinucleotide repeat expansions (> 39) in the ATXN1 gene. These mutations induce misfolding of polyQ-expanded ataxin-1, leading to its accumulation into toxic intranuclear inclusions in human neurons [23]. The exact mechanism of protein aggregation remains unknown. However, recent evidence indicates that abnormal PTMs in ataxin-1, especially phosphorylation, significantly accelerate the aggregation process [24]. Proteomics analysis in a cellular model of polyQ-expanded ataxin-1 aggregation enabled the construction of a perturbed hPIN [25]. The SCA1 hPIN network contained 12 out of 32 predicted PTM-PPIs directly related to common upstream regulators. A compact cluster composed of ataxin-1, its dysregulated PTM-PPIs and their upstream regulators highly correlated to SCA1, suggesting that it might represent a crucial part of disease pathology.

## Materials and methods

### Human protein interaction network construction

The hPIN is a subset of release 2.3 of the Human Integrated Protein–Protein Interaction rEference (HIPPIE; (26,27)). HIPPIE retrieves interactions between human proteins from major expert-curated databases and calculates a score for each one, reflecting its combined experimental evidence. The raw version of this network is available in the Download section of the HIPPIE database. In this study, the hPIN was constructed using interactions with confidence score ≥ 0.71 (selects for a high percentage of interactions supported by at least two publications [17]). After discarding self-interactions and extracting the network’s largest connected component (LCC) we obtained an hPIN consisting of 15,587 proteins (nodes; S1 Table) with 186,196 interactions (edges; S2 Table).

### Mapping the human protein interactome in hyperbolic space

We embedded the hPIN in the two-dimensional hyperbolic plane using the R package “NetHypGeom”, which implements the LaBNE + HM algorithm [28]. This algorithm combines manifold learning and maximum likelihood estimation to model the geometry of complex networks [29,30]. The PS model has a geometrical interpretation in hyperbolic space (H^2^) where nodes that join the system connect with the existing ones that are hyperbolically closest to them [12,14,30]. The network was embedded in H^2^ to infer the hyperbolic coordinates of each protein, with parameters γ = 2.97, T = 0.83, and w = 2π. The 15,587 nodes of the hPIN lie within a hyperbolic disc where the radial coordinate of a node, r_*i*_, represents the popularity dimension with nodes that joined the system first being close to the disc’s center. The angular coordinate, θ_*i*_, represents the similarity dimension [25–27].

### Clustering in the similarity dimension

To cluster proteins in the similarity dimension, we computed the difference between consecutive angular coordinates to identify big gaps. The nodes were sorted increasingly by their inferred angles *θ*, and the difference between *θ*_*i*_ and *θ*_*i + 1*_ was computed to identify the largest gaps between protein clusters in the similarity dimension. Gap size (g = 0.0077) that produces sectors with a minimum of three components, was chosen. The same process was followed to subcluster the proteins of the first sector, with a minimum of five components in each subcluster and gap size, g = 0.0042. (S1 Fig). To determine the start and the end of each cluster, we chose gap sizes *g* that produced clusters with a minimum number of members (3 and 5 respectively) because this allowed us to perform meaningful functional enrichment analysis of each group of proteins. We carried out Gene Ontology (GO) enrichment analysis for the proteins in each sector of the hPIN, using the nodes of the network as background set. Only GO Biological Process (BP) terms enriched at a signiﬁcance level (p-value) of 0.05 or less were kept. Neighboring clusters with similar biological functions were merged to avoid redundancy.

### Selection of experimentally known phosphorylation and dephosphorylation PPIs

The functional associations for the interactions within the hPIN were extracted from multiple providers using the PSIQUIC webservice [31]. PSIQUIC enables access to molecular interaction databases supporting the PSI-MI format, which provides a hierarchical structure describing protein interactions. Specifically, we considered the interactions annotated with the children terms of the PSI-MI category 0414 *“enzymatic reaction”* and particularly we focused on two of them: PSI-MI category 0217 *“phosphorylation reaction”* and 0203 *“dephosphorylation reaction”*. The frequency of use of other terms (e.g., ubiquitination, methylation, acetylation) were too low for our purposes.

From the interactions annotated as phosphorylation or dephosphorylation, we selected those for which we were able to determine the direction of PTM activity from an effector protein (protein kinase or protein phosphatase) to a target according to the annotations of the interacting proteins. To identify effector proteins we used KinaseMD [32] and the human DEPhOsphorylation Database (DEPOD) [33]. We discarded cases in which neither protein was identified as a putative effector, or both proteins were identified as one effector type (protein kinase or protein phosphatase), because it is not possible to identify the direction of the PTM-related interaction in these. We obtained a total of 295 PPIs as PTM-related directed interactions (from effector protein to target protein; training dataset; S3 Table). Two cases involved a protein kinase and a protein phosphatase mutually acting on each other. These 295 PPIs were used as positives and the rest were used as negatives to train our model.

### Feature extraction

We used a total of 14 features to train a classifier to detect PTM-related directed PPIs. Given a directed PPI to test, one node is taken as effector and the other as target according to the direction being tested. Six properties are taken for effector and target: two are their hyperbolic coordinates (r and theta), and the other four are measures of centrality. In network analysis, centrality measures evaluate the importance of a node based on certain parameters [34]. As measures of centrality, we used degree centrality (DC), betweenness centrality (BC), closeness centrality (CC) and eigenvector centrality (EC). DC is the number of immediate neighbours of a given node [35]. BC computes the significance of a node by calculating the fraction of all shortest paths that pass through it [36]. CC defines the proximity of a node to all the others [37] and EC reflects the influence of a node in a network [38]. The remaining two properties are defined for the edge: hyperbolic distance between the interacting proteins and r difference (absolute value). The values used are available in S1 and S2 Tables.

### Model development and evaluation

Model developing was done using the “caret” package in R [39]. For the primary model building we used k-fold repeated cross validation in a training partition (70%) and validated the model in a leave out external validation sample (30%). We used the random forest (RF) algorithm [40] from the “caret” package to train our model. Five-fold repeated cross-validation was used (*repeats* = 10) to identify optimal hyperparameters. The parameter values were varied, and optimal values were chosen based on the accuracy (*mtry* = 14, *ntrees* = 500). We report accuracy scores in the 5-fold repeated cross validation (*repeats* = 10) samples. To address class imbalance, we used the under-sampling technique while sampling for cross validation. The importance of each feature was then calculated. This study implemented a ROC curve to determine the efficacy of the RF model. The receiver operator curve (ROC) represents the relationship between false positive rate and the true positive rate in a plane for each cut-off value used to define positive and negative classification results [41]. We then calculated the area under the curve (AUC) value, which describes the classifier’s ability to discriminate between positive and negative results. It is a standard measurement of prediction quality and is commonly used to compare performance of models [42].

### Comparison of predictions by alternative methods

We used kinase-substrate predictions by two alternative methods to add support to our predictions: PhosD [43] and Phosformer-ST [44]. We obtained a set of predictions from PhosD using a score threshold of 0.5 (1852 predictions). Of those, only 1062 overlapped with PPIs in our HIPPIE dataset. Phosformer-ST assigns scores to serine/threonine phosphorylation sites. To be able to compare this approach with ours, we re-assigned the predictions at a protein level, indicating as phosphorylated proteins that contain at least one peptide with a score above 0.5. Isoforms were removed from the comparison set, since the tools’ predictions are based on sequence fragments, and they can diverge among isoforms. This resulted in a set of 451,724 predictions. Of those, only 961 overlapped with PPIs in our HIPPIE dataset.

### Mass spectrometry (MS) analysis

The generation of Tet-On YFP-ATXN1(Q82) mesenchymal stem cells (MSCs) has been previously described [45]. Cells were cultured for 10 days in the presence or absence of doxycycline. For protein extraction and solubilization, technical triplicates of cells were vigorously shaken at 95 °C with hot SDT buffer and centrifuged at high speed. Protein solutions were loaded into a polyacrylamide gel and stained by Coomassie Brilliant Blue G-250 (CBB-G250) for sample quality control. A cut-off filter of 10 kDa was used for FASP sample processing, which includes protein reduction by dithiothreitol and alkylation by iodoacetamide to prevent disulfide bond formation following incubation of the samples in presence of trypsin at 37 °C for 18 hours. Extraction with ethyl acetate solvent was used for the removal of any potential SDS traces from the resulting peptide mixture. Liquid Chromatography with tandem mass spectrometry (LC-MS/MS) analysis of peptide mixture was performed using the Ultimate 3000 RSLCnano system (Thermo Fisher Scientific) followed by the Orbitrap Q-Exactive HF X system (Thermo Fisher Scientific). The analytical column outlet was linked to the Digital PicoView 550 (New Objective) ion source, coupled with the Active Background Ion Reduction Device (ABIRD, ESI Source Solutions). MS data were acquired in a data-dependent strategy selecting up to the top 20 precursors.

### MS data processing

Raw data obtained from MS were processed on MaxQuant (version 1.6.3.3) with Andromeda search engine utilization. Peptide sequences were annotated on the UniProtKB database (version 20180912, Human) and MaxQuant contamination databases (downloaded with the given version). Mass tolerances for peptides and MS/MS fragments were 4.5–10 ppm and 0.05 Da, respectively. Oxidation of methionine, deamidation (N, Q) and N-terminal acetylation were set as optional protein modifications, while carbamidomethylation (C) was set as fixed protein modification. Two enzyme miss-cleavages were permitted for the final annotation. Peptides and proteins with false discovery rate (FDR; q-value) < 1% were considered. The MaxQuant label-free quantification algorithm (MaxLFQ) was applied for global data normalization (minimal ratio count 1) and the MaxQuant protein group list was further analyzed via KNIME Analytics Platform (v.3.7.1). Results were deposited in the PRIDE Archive (https://www.ebi.ac.uk/pride/archive, accession number: PXD038393).

### Construction of SCA1 PPI networks and enrichment analysis

Proteins were annotated in the HIPPIE database for the retrieval of high-confidence protein-protein interaction (PPI) scores ( ≥ 0.71). A PPI network was constructed in Cytoscape [46] and proteins were further clustered in functional communities using the GLay algorithm [47]. The layout of the network was designed in the Gephi platform [48]. For the prediction of upstream regulatory kinases, proteins were annotated in the X2K Appyters platform using the KEA3 database [49]. Predicted kinases were filtered with an overall score lower than 75. Over-representation analyses for KEGG biological pathways, human diseases (Jensen diseases), proteomics signatures (ProteomicsDB) and cell types and tissues (Descartes) were performed using the EnrichR package [50].

### Drug repurposing and repositioning

Protein lists were uploaded in the L1000FWD platform [51] according to their pattern of dysregulation and hits were sorted based on their combined score. Mechanism of action and drug target identification were studied on the PHAROS, DrugBank and Reactome platforms. Evidence for drug safety and usage was obtained from the International Clinical Trials Registry Platform.

## Results

### Prediction of phosphorylation and dephosphorylation directed PPIs

**Fig 2:**
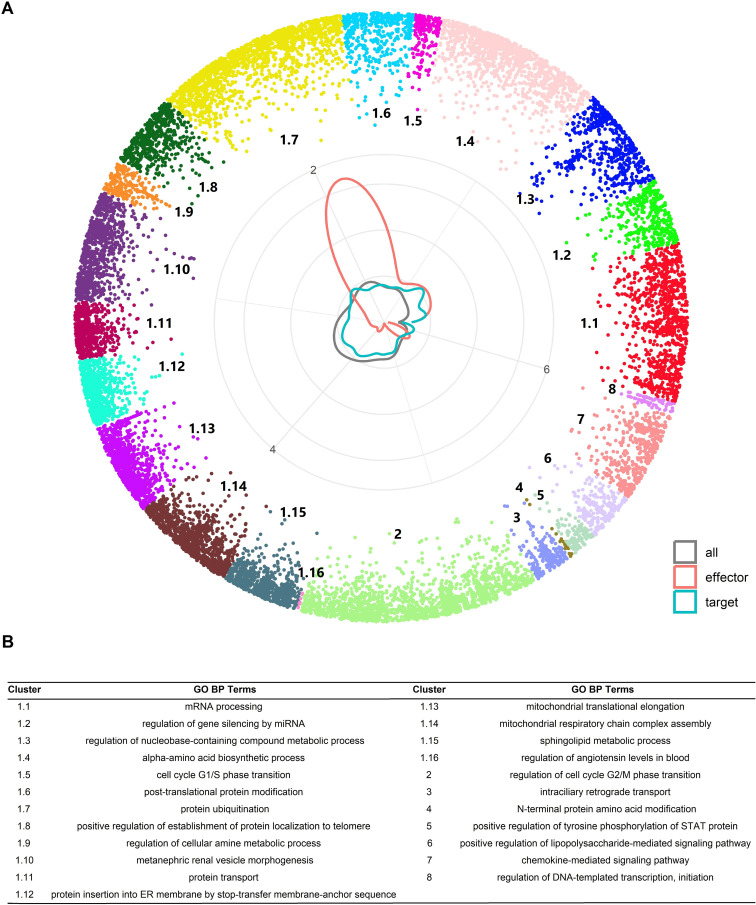
Properties of the hPIN. (A) Positions of nodes. Colors indicate node clusters. The circular plot at the center indicates the density of nodes in the angular dimension for all nodes (gray), and for the nodes of 295 directed PTM-PPIs with experimental evidence used for training a predictor: effectors (red) and targets (blue) (see text for details). (B) Top enriched GO BP term for each cluster.

To predict PTM-related directed PPIs (PTM-PPIs) we considered the entire dataset of human PPIs mapped in hyperbolic space (hPIN; see Methods for details). In this space, the angular coordinates (theta) represent the similarity between the nodes in terms of interacting partners, and shorter distance to the center (r) corresponds to nodes with higher connectivity. The angular coordinate of the nodes in the hyperbolic plane reflects characteristics that make a node similar to the others. From a biological point of view, proteins agglomerate in the angular dimension of the H^2^ capturing functional organization [15,17,52]. To investigate the biological meaning of the theta coordinates, we find proteins grouped in clusters by identifying gaps between consecutive inferred angles (S1 Fig, see Methods for details). This resulted in 24 clusters in the hPIN. The proteins are grouped in a similarity-based manner as each cluster is found to be enriched with various aspects of the GO biological process (Fig 2).

To predict PPIs as directed PTM-PPIs we collected a dataset of 295 experimentally supported interactions involving protein phosphorylation or dephosphorylation. We selected PPIs for which one of the interacting proteins is a putative effector (kinase or phosphatase) while the other is not (see Methods for details). We assume that this gives us a good estimate of the directionality of the interaction. Interestingly, the distribution of nodes corresponding to effectors and targets is different from that of the background proteome in the hPIN (Fig 2A). For example, effectors and targets appear to be depleted (less frequent than background) in clusters 1.13 and 1.14 associated with mitochondrial functions, while targets are enriched in cluster 1.1 associated with mRNA processing and effectors in cluster 1.7 associated with protein ubiquitination (Fig 2B). These results suggest that the hPIN provides information that could be used to discriminate effectors and targets of protein phosphorylation or dephosphorylation.

We chose 14 features to train a random forest (RF) model, six of them assigned to each of the two interacting nodes (r and theta coordinates in the hyperbolic map and four measurements of centrality), and two regarding the edge (hyperbolic distance and radial difference between the nodes) (See Methods for details).

**Fig 3:**
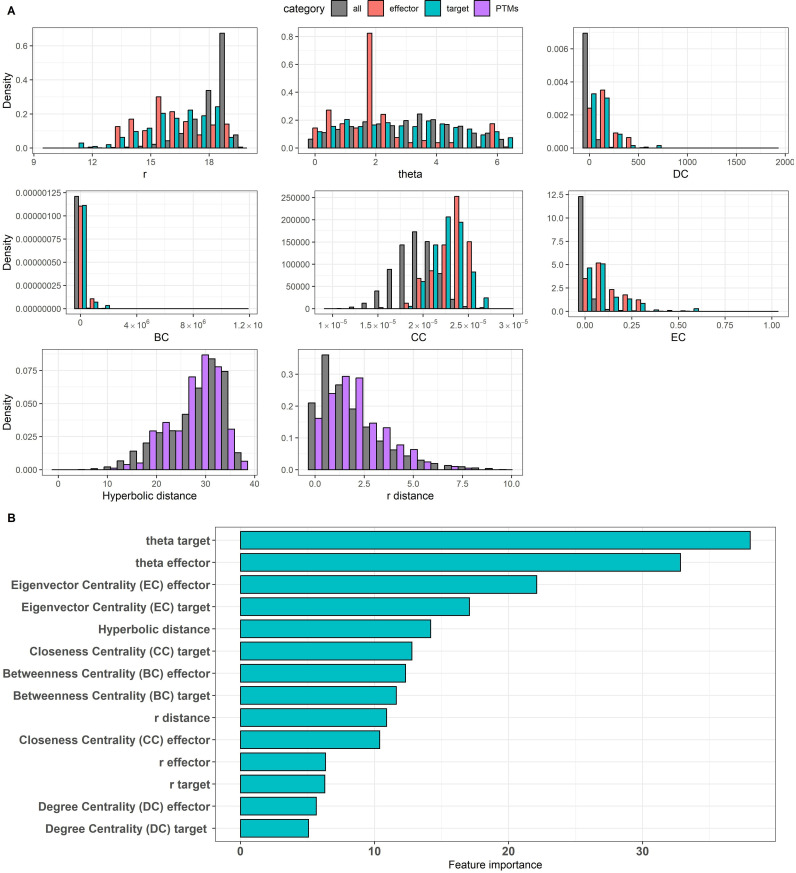
Properties of the 14 features that were used for building the model. (A) Distributions of r, theta, degree centrality (DC), betweenness centrality (BC), closeness centrality (CC) and eigenvector centrality (EC), hyperbolic distance and r distance. (B) Feature importance values indicate the impact of each predictor on the prediction model. For this study, the angular coordinates of the of effector and target proteins in the hyperbolic space are the most highly significant predictors of the model, confirming the importance of the embedding of the hPIN in H2.

Regarding these features, we could appreciate significant differences in their distributions for effectors, targets and background (Fig 3A). Regarding hyperbolic coordinates, the effectors of the 295 positive directed-PPIs had in general shorter radius than the background proteins. This was also the case for targets, although with a less pronounced difference. This indicates that targets and effectors are more interconnected than other proteins, which agrees with their contribution to signaling pathways, stronger for the effectors. Regarding the angular dimension, both effectors and targets have maxima at a position different to the background, with effectors having a more pronounced grouping around theta = 1.8, which corresponds to cluster 1.7 (see also circular plot in Fig 2).

Regarding centrality measures, targets and effectors have higher values than background. For the distributions with maxima at zero values of EC, BC and DC, targets seem to have a larger number of low values than effectors. For the Gaussian distribution of CC, again effectors have slightly higher values than targets. Together with the observations of shorter radius this is in accordance to the higher connectivity of effectors and targets, with effectors slightly more connected and central than targets.

Regarding the edges, the radius differences between the connected nodes are higher in PTM-PPIs than background. This suggests that these interactions have a greater capacity to connect highly and lowly connected regions of the hPIN. The hyperbolic distances between nodes are slightly lower than for background PPIs. This could reflect that these PTM-PPIs participate in closely connected pathways and signaling networks.

To train the RF, we used 70% of the 295 interactions with 5-fold cross-validation, while the remaining 30% were used as the validation set. We performed the cross validation independently 10 times. The model with the highest accuracy was chosen as the final prediction model and it was validated on the test set, showing an accuracy of 74% (S2A Fig; see Methods for details). A random forest (RF) classifier with 500 trees was able to produce satisfactory results. Finally, 74% sensitivity and 80% specificity were calculated from the confusion matrix. The receiver operating characteristic curve (ROC) had an AUC value of 0.87, indicating that the classifier could effectively find directed PTM-related PPIs based on topological and network properties of the interacting partners in the hPIN (S2B Fig). Additionally, the Precision-Recall curve (S2C Fig) was computed to assess the model’s performance in the context of the imbalanced dataset, providing further insights into the classifier’s ability to correctly identify the minority class (directed PTM-related PPIs).

Regarding the contribution of the features to the predictions, we observed that the angular coordinate of the target is the most important feature, closely followed by the angular coordinate of the effector (Fig 3B). The high relevance of the angular coordinates of effectors and targets is related to the fact that the angular positioning of the hyperbolic mapping captures the functional organization of the proteins in the cell.

EC of effector and target (which represents the importance of a node based on the links to important nodes) were the next features in order of importance. These were followed by the hyperbolic distance between the pairs of interacting proteins.

The next feature in order of importance was the CC of the target (which seemed to be much more important than that of the effector). This feature measures the central position of the node with respect to the entire network. The next features were the BC of the effector and of the target (representing how often a node is on paths between other nodes). We then have the r difference, the CC of the effector and, with much less importance, the r of the effector and of the target, suggesting that the distance to the center (which reflects the evolutionary age of the protein) is not very informative. Finally, the least important features are the DC values of the target and of the effector, which represent how well a node is directly connected to most nodes in the network.

It is interesting that the values of theta, and more marginally, the hyperbolic distance and the r distance between the nodes of PPIs were relevant features for the prediction. These results indicate that the embedding of the hPIN in hyperbolic space, which assigns these r and theta values to each node, can be useful to identify PTM-directed protein interactions. In particular, the contrast of the distributions of theta values of effectors and targets in the training dataset with the functions enriched in the corresponding clusters is revealing (see Fig 2A). While the maximum accumulation of effectors happens in a region of cluster 1.7 associated with the GO term “*protein ubiquitination*”, a wider maximum happens for effectors and targets around clusters 1.1–1.3 enriched with terms “*mRNA processing*”, “*regulation of gene silencing by miRNA*” and “*regulation of nucleobase-containing compound metabolic process*”. The latest includes the synthesis of DNA and RNA. The distribution of targets is more similar to the background than that of the effectors, suggesting that PTM regulation targets all cell processes. Differently, effectors have a tendency to occupy tighter angular regions of the map, suggesting an association with regulatory mechanisms of control. The association with protein degradation (ubiquitination) seems to be a salient feature, which agrees with mechanisms known to stop active kinases [53]. These distributions of theta values have biological significance, which explains why they had the best predictive value.

Regarding the centrality measures, which are independent of the hyperbolic mapping, it can be seen that while EC, particularly of the effectors, plays an important role, DC does not seem to contribute so much. In any case, all features receive non-null values, suggesting that they all have predictive value.

To get a better understanding of how important are the features for the predictions of (de-) phosphorylation directed PPIs, we trained five RF models masking different features each time and we evaluated the overall contribution of the attributes in terms of information gain. More specifically, we built the model on the 14 features and we determined the significance of each variable in the predictions using the *varImp ()* function from the random forest classifier. This method tracks the changes in model statistics for each predictor and accumulates the reduction in the statistic when each predictor’s feature is added to the model. This total reduction is used as the variable importance measure. We conducted the prediction process while masking different features and we observed the changes in the performance metrics of the model. We created five datasets, starting with 14 variables and then we removed them one by one in order of their importance (Fig 3B). ROC curves analysis of the different datasets showed that using 14 features, AUC has the higher value; while removing them, the AUC is reducing (S3 Fig). This finding indicates the importance of the hyperbolic properties and centrality measures in classifying the directed (de-) phosphorylation related PPIs. The prediction model was applied to the entire set of edges. As we predict directional PTM-PPIs (with an effector and a target) all edges were tested in both directions for a total of n = 2 x 186,198 = 372,396 evaluations. The model produces a probability of the interaction being a directed PTM-PPI or not. A total of 117,655 directed interactions received a score>= 0.5 and 6,790 a score>= 0.9 (S4 Table).

The table of predictions allows easily to find the best predictions as target or effector for every protein in the network. For example, MAPK3 (MAP kinase-activated protein kinase 3; UniProt ID MAPK3_HUMAN) has a total of 9 edges; none of them were part of the experimentally verified set used in the training. Regardless, the predictions make sense: 8/9 have a score>= 0.5 for MAPK3 as effector (the best one is for HSPB1 as a target; score = 0.942), and there is only one with a score>= 0.5 for MAPK3 as target (with PRKY; PRKY_HUMAN), which is precisely the one with score < 0.5 for MAPK3 as effector. PRKY is a putative serine/threonine protein kinase with very little experimental information and its prediction as effector over MAPK3 is modest (score = 0.59), but the fact that it is annotated as protein kinase makes the prediction plausible.

We see that the classifier has trouble assigning the correct direction of the interaction. For example, the edge CHK2 (CHK2_HUMAN) RB (retinoblastoma; RB_HUMAN), which was used as positive for training, is highly evaluated with RB as target (score = 0.968), but also with RB as effector (score = 0.78). This indicates that the predictions need to be taken with care but also suggests that the scores can be compared.

**Fig 4:**
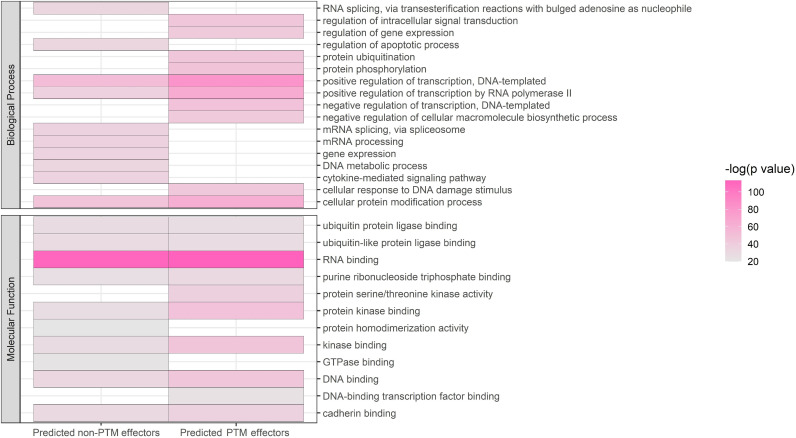
Enrichment analysis of predicted effectors. Left, negative predictions. Right, positive predictions. Colors from pink to grey indicate p-values from high to low; a lower p value suggests the proteins are more enriched in GO Biological Process and Molecular Function terms related to post-translational modifications. GO BP and MF terms are more enriched in the PTMs class, which supports the good performance of the model.

To evaluate whether our predictions collectively are meaningful from a biological point of view, we performed a Gene Ontology (GO) enrichment analysis of proteins predicted even once as effectors (n = 12,115, Fig 4). Even considering that this is a large number of proteins, regarding GO Biological Process terms, these proteins are enriched in terms such as *“ubiquitin-dependent protein catalytic process”*, *“protein ubiquitination*”, *“protein phosphorylation”*, and “*cellular protein modifications*”. Additionally, GO Molecular Function terms like *“protein serine/threonine kinase activity”*, *“protein kinase binding”* and *“kinase binding”* are also enriched. For comparison, we computed the enrichment for proteins predicted even once as not being effectors (n = 13,784; the two lists overlap in 10,314 proteins) and most of these terms received less significant p-values. This functional analysis supports the good performance of our prediction model.

### Support of the predictions by other methods

To add support to the predictions of our model, we verified which of our predictions were detected by two alternative approaches: PhosD [43] and Phosformer-ST [44] (see Methods for details). PhosD is a kinase-substate prediction tool based on protein domains. Phosformer-ST is a machine learning tool that uses serine/threonine phosphorylation sites comprised of 15-mer peptides, assigning scores for these regions. A total of 788 and 535 predictions were supported by PhosD and Phosformer-ST, respectively, with an overlap of the three methods for 24 predictions. The detailed prediction results can be found in S4 Table.

### Proteomics analysis highlights dysregulated biological pathways in a SCA1 cellular model

The results presented above suggest that the hyperbolic mapping of the hPIN provides a predictive value for direct PTM-PPIs. However, the interpretation of individual prediction scores remains complex. Therefore, we hypothesized that these predictive insights can collectively help us understand perturbations of the hPIN, which could be particularly valuable in identifying therapeutic mechanisms for human diseases. This is especially relevant for complex neurodegenerative diseases, in which the normal interactome is reportedly disrupted and abnormal PTMs can promote a plethora of pathological events. To test this hypothesis, we analyzed proteomics data from a SCA1 cellular model, in which the hPIN is perturbed due to the accumulation of inclusions of polyQ-expanded ataxin-1.

In particular, proteome alterations driven by the accumulation of mutant ataxin-1 were studied in Tet-On YFP-ATXN1(Q82) MSCs, a previously characterized cellular model of protein aggregation [45]. These cells contain insoluble intranuclear inclusions of polyQ-expanded ataxin-1 with a β-sheet conformation, an event that characterizes late-stage SCA1. Global proteome profiling was performed in inclusions-containing cells (SCA1, n = 3) and control cells (CTL, n = 3; see Methods for details). As a result, 3,926 proteins were identified and 3,179 of them were quantified in all six samples. The two conditions were efficiently discriminated by principal component analysis, using as a criterion the variance in protein representation in each group (S4A Fig).

To create a representative protein network for SCA1 cellular pathology, we filtered 805 dysregulated proteins by a | log2 FC | ≥  0.5 and adj. p-value ≤ 0.05 (S4B-C Fig) and retrieved their high confidence interaction scores using the HIPPIE platform [26,27]. As a result, a complex PPI network of 636 significantly dysregulated proteins was generated, representing proteome alterations due to the accumulation of polyQ-expanded ataxin-1 inclusions (S5 Table).

The PPI network was further divided into smaller communities of densely interacting proteins, which outline functional modules (see Methods for details). Ataxin-1 was detected in the largest community (C1), which was highly associated with neurodegeneration and neuronal-related terms (S5A Fig). Enrichment analysis for biological pathways on the next 4 largest communities revealed a significant implication of spliceosome, lysosome and ribosome, as well as metabolic pathways (C2-C5, respectively) (S5A Fig). Interestingly, clustering and analysis of a control PPI network generated from a randomly selected protein dataset (n = 805) did not result in similar enrichment terms, indicating that the SCA1 PPI network and sub-communities are not generic but strongly associate with polyQ aggregation.

### PTM-PPIs of ataxin-1 are components of the SCA1 network

To date, the effect of PTM-PPIs on the aggregation of mutant ataxin-1 remains unknown. Therefore, we sought to identify potential PTM-PPIs of ataxin-1 which may be involved in polyQ protein aggregation and eventually SCA1 pathogenesis. In the SCA1 PPI network, ataxin-1 directly interacted with 21 proteins. Implementation of our algorithm suggested that 13 of them may have a post-translational modification activity. Specifically, four of these proteins (gene names: *ANP32A*, *EIF3F*, *GSPT1* and *USP7*) were downregulated, while nine of them (gene names: *DNAJB6*, *HSPB1*, *PHPT1*, *SNCA*, *SQSTM1*, *SUMO1*, *TBL1XR1*, *TRIP6* and *TPM3*) were upregulated in SCA1 cells (S6 Table). The enzymatic activity of phosphohistidine phosphatase 1 (*PHPT1*), small ubiquitin-related modifier 1 (*SUMO1*), Ubiquitin-specific-processing protease 7 (*USP7*) and the chaperone proteins (*SNCA*, *HSPB1* and DNAJB6) have been previously described, while no such information exists for the rest (7/13) of the predicted proteins [54–57]. PTM-PPIs of ataxin-1 mainly clustered in community C1 (associated with neurodegeneration, eight proteins) and to a lesser extent in C2 (associated with spliceosome; four proteins) and C4 (associated with lysosome; one protein) (S5A Fig).

In an attempt to find a link among the three major communities containing the PTM-PPIs of ataxin-1 (C1, C2 and C4), we searched for potential common upstream regulators. To do so, the proteins of each cluster were considered substrates and were annotated using the KEA3 database for the prediction of regulatory kinases (see Methods for details). According to the results, 21 kinases were identified as potential common regulators for all three communities. Interestingly, three of them (*MAPK1*, *MAPK3* and *CDK4*) were indeed significantly dysregulated in SCA1 cells (S6 Table), suggesting their potential impact on regulating the C1, C2 and C4 communities. These kinases interact with various components of clusters C1 and C2 but not with proteins of C4, while none of them directly interacts with ataxin-1 (S5B Fig). Remarkably, no significantly dysregulated kinases were identified when repeating the analysis for a randomly sampled test PPI network, underscoring the specificity of the identified kinases to the SCA1-related network.

### Identification and restoration of critical components of the SCA1 PPI network

**Fig 5:**
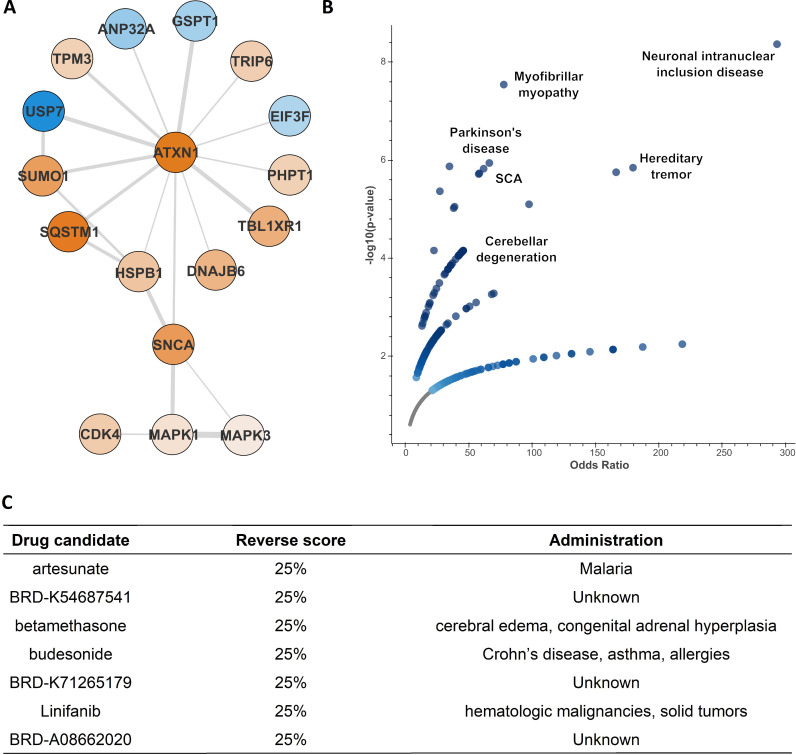
Critical proteins associated with SCA1 pathology. **(A)** A PPI network consisting of ataxin-1, its 13 PTM-PPIs and the 3 dysregulated kinases (n = 17). Node color corresponds to fold change (blue for down- and orange for upregulated proteins, respectively) and edge width to HIPPIE interaction score [24]. **(B)** Enrichment analysis for rare diseases highlighted an association of the critical proteins with SCA-related terms. **(C)** Known drugs which may restore the levels of at least 25% of the dysregulated proteins, ranked according to their combined score (p-value, adj. p-value, z-score).

SCA1-related cellular pathology might be driven by a few specific components scattered within the disease PPI network. To address this hypothesis, we generated a sub-network consisting of ataxin-1, its predicted PTM-PPIs (n = 13) and their three common upstream kinases (MAPK1, MAPK3 and CDK4) (S6 Table; see Methods for details). Interestingly, all three identified kinases were connected to ataxin-1 through α-synuclein (*SNCA*), a protein associated with several neurodegenerative diseases and particularly Parkinson’s disease (Fig 5A). Enrichment analysis for rare diseases (see Methods for details) indicated that this sub-network is associated with cerebellar degeneration terms, including Spinocerebellar Ataxia and the formation of nuclear inclusion bodies. This result suggests that these proteins are critical for the disease and their dysregulation may underlie SCA1-related pathological events (Fig 5B).

Therefore, restoration of their dysregulation pattern might mitigate disease progression. To this end, we searched for candidate drugs potent to increase the levels of downregulated proteins and decrease those of upregulated ones. Their reverse score indicated the overlap between the input proteins and the altered signature after drug administration. Hits with at least a 25% reverse score were considered significant candidates (see Methods for details). Then, they were sorted by descending combined score, considering the reverse score, p-value and Z-score. From this analysis, we identified four known drugs (artesunate, linifanib, budesonide and betamethasone) and three novel compounds (BRD-K54687541, BRD-K71265179 and BRD-A08662020) as potential treatment approaches (Fig 5C). These agents might mitigate polyQ-expanded ataxin-1-associated neuropathology in SCA1 cells potentially leading to the development of novel therapeutic strategies against the disease.

## Discussion

Machine learning has shown good performance in extracting rules from massive biological data. Here we present a computational method that implements machine learning based on the random forest algorithm and trains a model to predict directed PTM-PPIs, concretely, phosphorylation and dephosphorylation interactions between a target and an effector. Several lines of work approach PPI prediction through various computational methods [58–64] but currently not much research has been performed on predicting the function of PPIs. The representation of the human protein interaction network in the two-dimensional hyperbolic plane has been shown to be both meaningful and useful: inferred node coordinates uncover information about protein evolution and function, whereas hyperbolic distances can be used to identify potential protein interactions [15]. In this study, we report another scenario, in which hyperbolic properties together with metrics from network analysis are used to predict directed PTM-PPIs.

The result that the angular (theta) coordinates of targets and effectors were the more predictive features is of particular relevance, considering that they are superior to network measures that are not depending on the hyperbolic mapping. The fact that theta of target is more predictive that than that of the effector is consistent with targets being responsible of narrower functions (signaling, cell cycle control, cell differentiation), while effectors, with a more upstream position on a regulatory network, could be expected to have more general functions, and therefore, less restrictions to take various angular positions in the hyperbolic map as core components of signaling cascades [65].

Our results evaluate various centrality measures suggesting that while all of them have predictive value, degree centrality may be the less informative compared to eigenvector, closeness and betweenness, at least, when evaluating phosphorylation and dephosphorylation. The complete list of predicted effectors turned out to be explainable from a biological point of view according to functional enrichment analysis.

To illustrate how to use the collective predictions for studying disease progression, we predicted PTM-PPIs in a SCA1 disease model with intense hPIN perturbation. The cell model employed here recapitulates key pathological features of SCA1, one of several polyQ diseases that are caused by expanded CAG repeats encoding a long polyQ tract in the respective proteins and lead to neurodegeneration [23,45,66].

Two possible factors contributing to selective neuronal impairment are the abnormal subcellular localization of polyQ proteins and the change of folding and function [67]. PTMs are shown to regulate properties including their intracellular localization and functions [68]. Consequently, understanding the effect of PTMs in polyQ diseases may yield important insight into mechanisms behind neuronal damage and more specifically in SCA1. We identified proteins that could be at the center of dysregulated phosphorylation networks and generated a disease-specific PPI network enriched with our PTM-PPI predictions. Among them, we identified α-synuclein (SNCA), a protein that translocates between the cytoplasm and the nucleus. SNCA may also function as a chaperone as it shares physical and functional homology with the 14-3-3 protein family which are responsible for ataxin-1 translocation from the cytoplasm to the nucleus [69–72]. Interestingly, SNCA is also involved in the pathogenesis of Parkinson’s disease and may impart its effect in line with the key upstream kinases of the SCA1 disease network [73].

To evaluate the translational impact of the predicted outcome, we searched for existing drugs that could revert the production of these proteins as potential therapeutics against the disease. By implementing a network-based drug repurposing analysis, we identified 7 potential drug candidates. Artesunate was the top hit, acting as a protein synthesis inhibitor and a glucocorticoid receptor agonist. We have previously shown that the accumulation of mutant ataxin-1 disrupts ribosome assembly and causes proteome instability [45,74]. Therefore, regulation of translation may have a therapeutic effect in SCA1 cells. Interestingly, artesunate is currently evaluated as a therapeutic agent for Friedreich’s ataxia (FA), suggesting that it might be also relevant for the treatment of other similar disorders, including SCA1 [75]. Furthermore, the predicted drug candidates betamethasone and budesonide also act as glucocorticoid receptor agonists. Although there is no direct evidence for SCA1, activation of glucocorticoid receptors seems to attenuate the aggregation of polyQ-expanded ataxin-3 and huntingtin proteins in SCA3 and Huntington’s disease (HD), respectively [76,77].

Our work supports the value of the hPIN and of their hyperbolic mapping for the prediction of the function of directed PTM-PPIs. The method was limited to detect phosphorylation and dephosphorylation, the most common PTMs, as directed interactions between a regulatory protein and its target; more complex interactions can be expected since regulatory proteins are often multi-domain proteins with a multiplicity of sites for their own regulation: our approach cannot be expected to capture those without an appropriate training dataset. In addition, we focused on phosphorylation and dephosphorylation, without making a distinction between them and, most importantly, without considering other less frequent but relevant PTMs such as ubiquitination, methylation or acetylation. Even within these limitations, we were able to apply our predictions to provide a proteome-wide set of scored interactions that we used to suggest therapeutic actions against a neurodegenerative disease. Our predictions should find applicability in combination with many other experimental and computational datasets.

## Supporting information

S1 Fig Identification of big gaps between inferred protein angles.
Proteins were sorted increasingly by their inferred angular coordinates θ and the difference between θi and θi + 1 was computed. The peaks correspond to gap sizes in the angular dimension and hint at the presence of similarity-based clusters. To determine the beginning and end of each cluster in the hPIN, we chose the gap size (g = 0.0077, line in red color) that produced clusters with a minimum of three components. The same process was followed to subcluster the first sector into 15 smaller clusters using a smaller gap size (g = 0.042, line in blue), This allowed us to perform meaningful enrichment analysis of each group of proteins.
(JPG)

S2 Fig Evaluation of the model.
(**A**) Accuracy for all the models after 5- fold cross validation repeated 10 times. (**B**) The ROC of the model confirms a satisfactory classification performance. (**C**) Precision-Recall curve, providing additional performance evaluation.
(JPG)

S3 Fig ROC curves and AUC scores to compare the classifier performance of the different data sets containing various number of features.
In total 14 features related to hyperbolic properties and centrality measures were used to predict phosphorylation and dephosphorylation directed PPIs.
(JPG)

S4 Fig Profile of dysregulated proteins in SCA1 cells containing polyQ inclusions.
**(A)** SCA1 cells were efficiently discriminated from control cells (CTL) using PCA. **(B)** Volcano plot depicting 449 significantly downregulated proteins (blue color) and 356 significantly upregulated proteins (red color) in SCA1 cells [selection criteria (log2FC ≤  | 0.5 | , adj. p-value ≤  0.05)]. The top 10 dysregulated proteins are highlighted in the plot. **(C)** Heatmap plot according to Euclidean distance indicates two distinct groups of up- and down-regulated proteins (red and blue color, respectively) in SCA1 and control cells.
(JPG)

S5 Fig Connectivity of dysregulated proteins in SCA1 cells.
**(A)** PPI network of significantly dysregulated proteins in SCA1 cells. Proteins were clustered into dense communities representing functional modules. Enrichment analysis on each cluster indicated a strong association with neurodegeneration (C1), spliceosomal (C2) and lysosomal (C4) activity, ribosome assembly (C3) and metabolic pathways (C5). Ataxin-1 directly interacts with 21 proteins, 13 of which are predicted as PTM-PPIs and participate in C1, C2 and C4. Both PTM and non-PTM PPIs of ataxin-1 are highlighted with red and yellow color, respectively. **(B)** Identification of regulatory kinases for C1, C2 and C4 clusters, which contain the PTM-PPIs of ataxin-1. MAPK1, MAPK3 and CDK4 are significantly dysregulated in SCA1 cells.
(JPG)

S1 Table Nodes of the hPIN. Columns indicate protein identifiers (UniProtKB), hyperbolic coordinates (r, theta), and centralities (Degree DC, Betweenness BC, Closeness CC and Eigenvector EC).
(XLSX)

S2 Table Edges of the hPIN.
Columns indicate protein identifiers (UniProtKB; p1, p2), hyperbolic distance, r difference.
(XLSX)

S3 Table Training dataset of experimentally known phosphorylation and dephosphorylation PPIs.
Columns indicate effector protein identifier (UniProtKB; p1), effector type, and target protein identifier (UniProtKB; p2).
(XLSX)

S4 Table Prediction scores of directed PTM-PPIs.
Columns indicate predicted effector and target protein identifiers (UniProtKB; p1, p2), score of the prediction of our method and classification of our method, PhosD and Phosformer-ST.
(XLSX)

S5 Table Significantly dysregulated proteins (|log2FC | ≤  0.5, adj. p-value ≤  0.05) in SCA1 (805 proteins).
Columns indicate protein identifier (UniProtKB ID and gene name), log2FC value, p-value, adjusted p-value and cluster number of the proteins SCA1 PPI network: values are (i) C1-C5 or unclustered for the 636 strongly connected proteins or (ii) blank for the remaining 169 less connected proteins.
(XLSX)

S6 Table Components of a critical PPI network involved in SCA1 pathogenesis.
Columns indicate protein identifiers (UniProtKB ID and gene name), log2FC value and adjusted p-value.
(XLSX)
