# Supplementary figures and images for: Prediction of protein interactions with function in protein (de-)phosphorylation
Source: PLoS One. 2025 Mar 3;20(3):e0319084. doi: 10.1371/journal.pone.0319084 (PMC11875375; doi:10.1371/journal.pone.0319084)

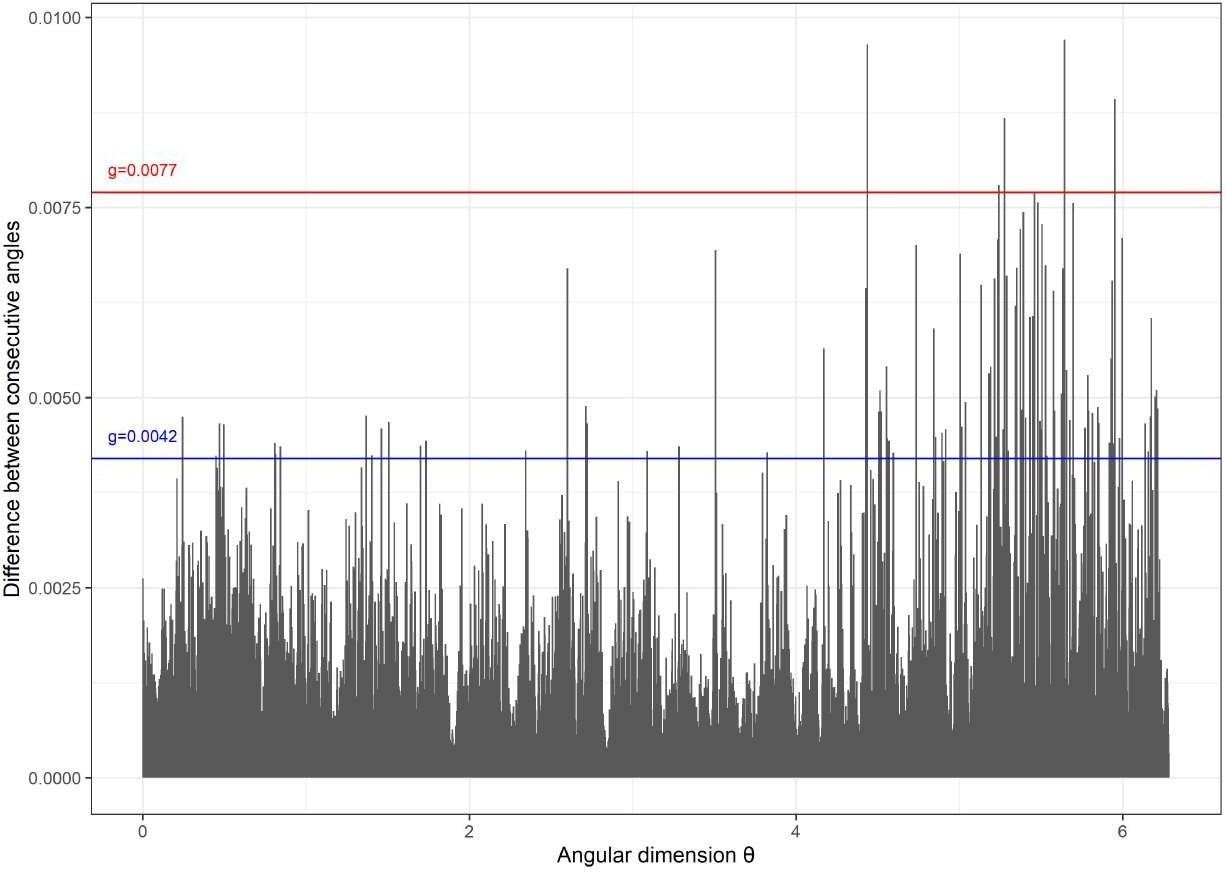

Supplement: S1 Fig — Proteins were sorted increasingly by their inferred angular coordinates θ and the difference between θi and θi + 1 was computed. The peaks correspond to gap sizes in the angular dimension and hint at the presence of similarity-based clusters. To determine the beginning and end of each cluster in the hPIN, we chose the gap size (g = 0.0077, line in red color) that produced clusters with a minimum of three components. The same process was followed to subcluster the first sector into 15 smaller clusters using a smaller gap size (g = 0.042, line in blue), This allowed us to perform meaningful enrichment analysis of each group of proteins. (JPG) [file pone.0319084.s001.jpg]

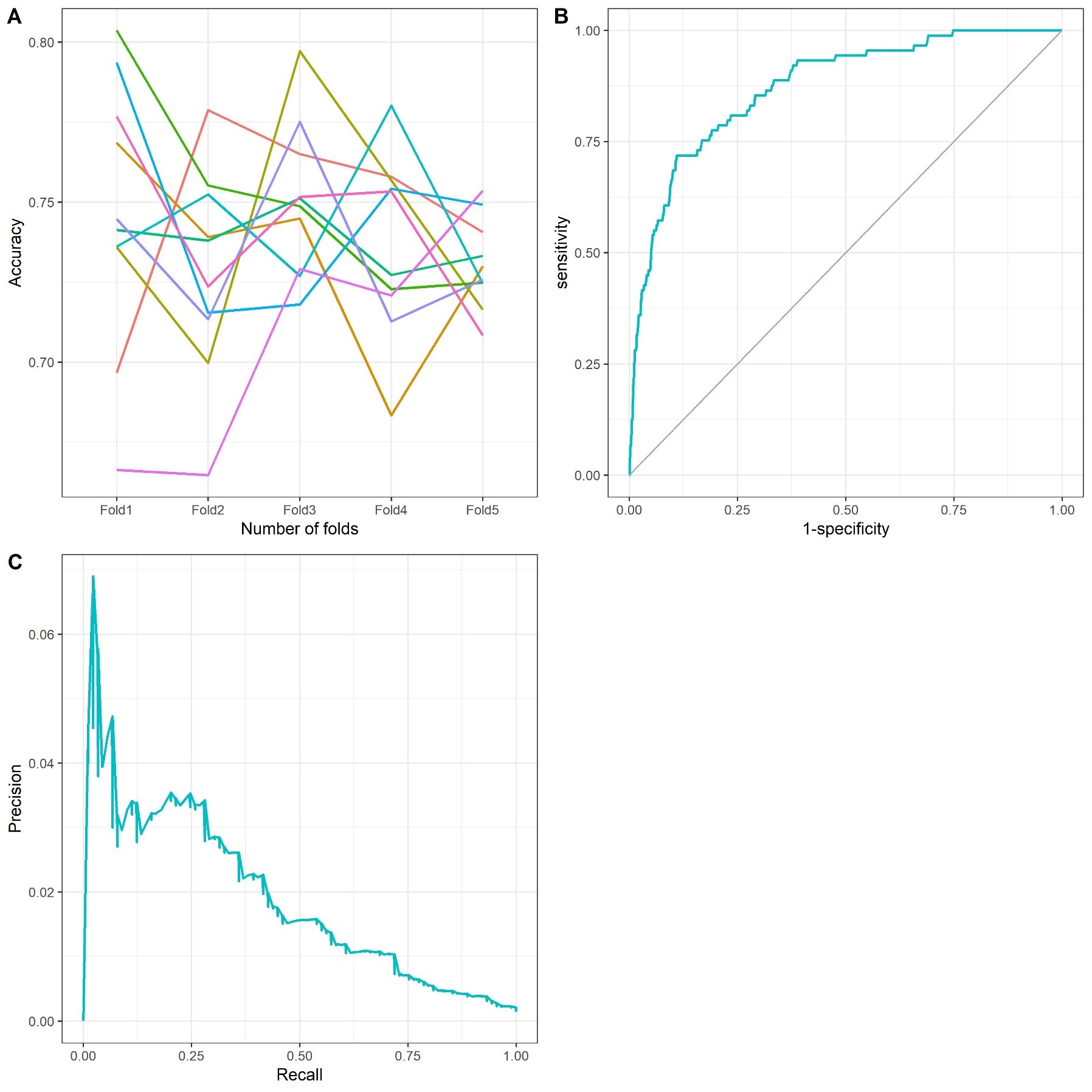

Supplement: S2 Fig — (A) Accuracy for all the models after 5- fold cross validation repeated 10 times. (B) The ROC of the model confirms a satisfactory classification performance. (C) Precision-Recall curve, providing additional performance evaluation. (JPG) [file pone.0319084.s002.jpg]

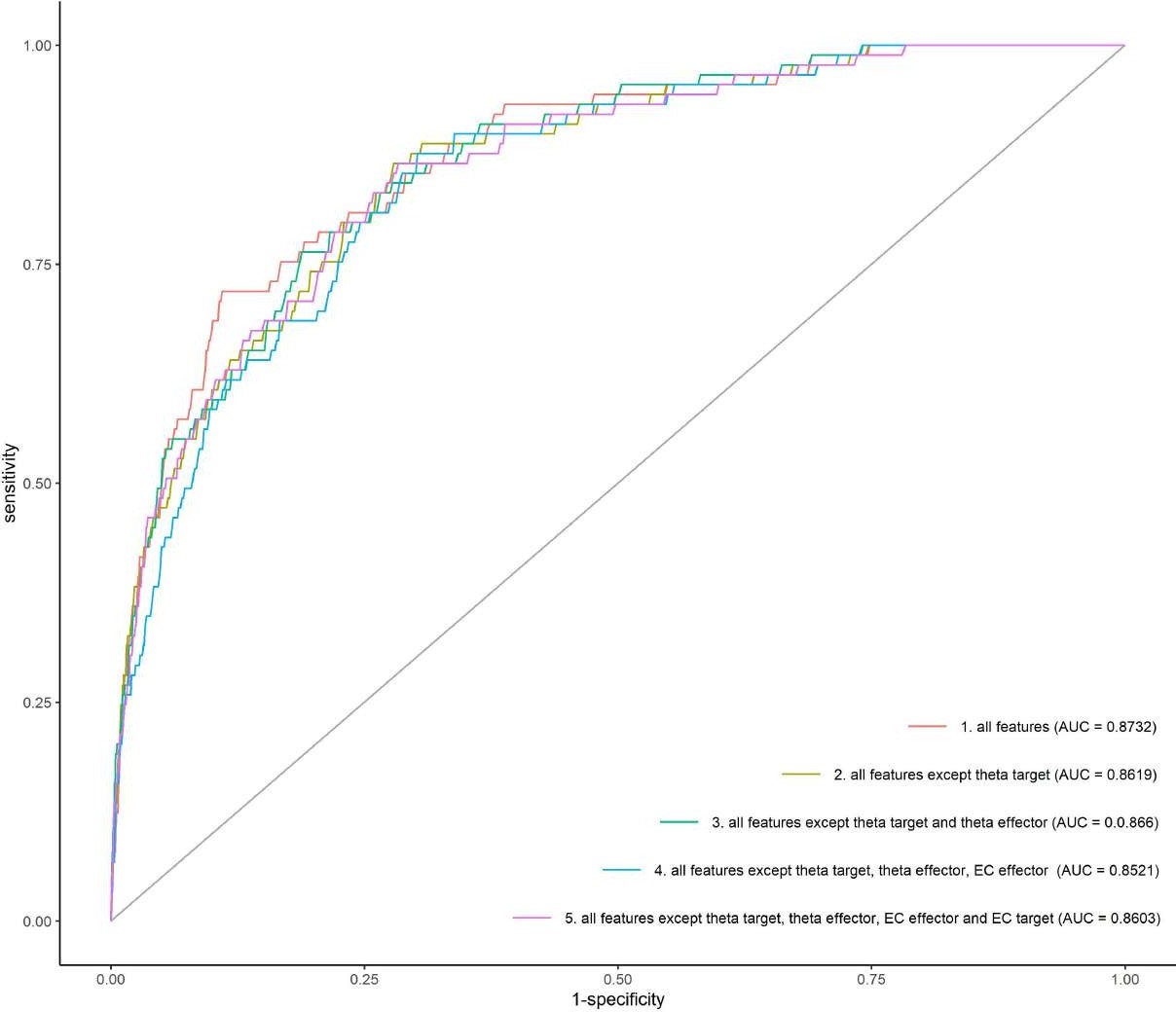

Supplement: S3 Fig — In total 14 features related to hyperbolic properties and centrality measures were used to predict phosphorylation and dephosphorylation directed PPIs. (JPG) [file pone.0319084.s003.jpg]

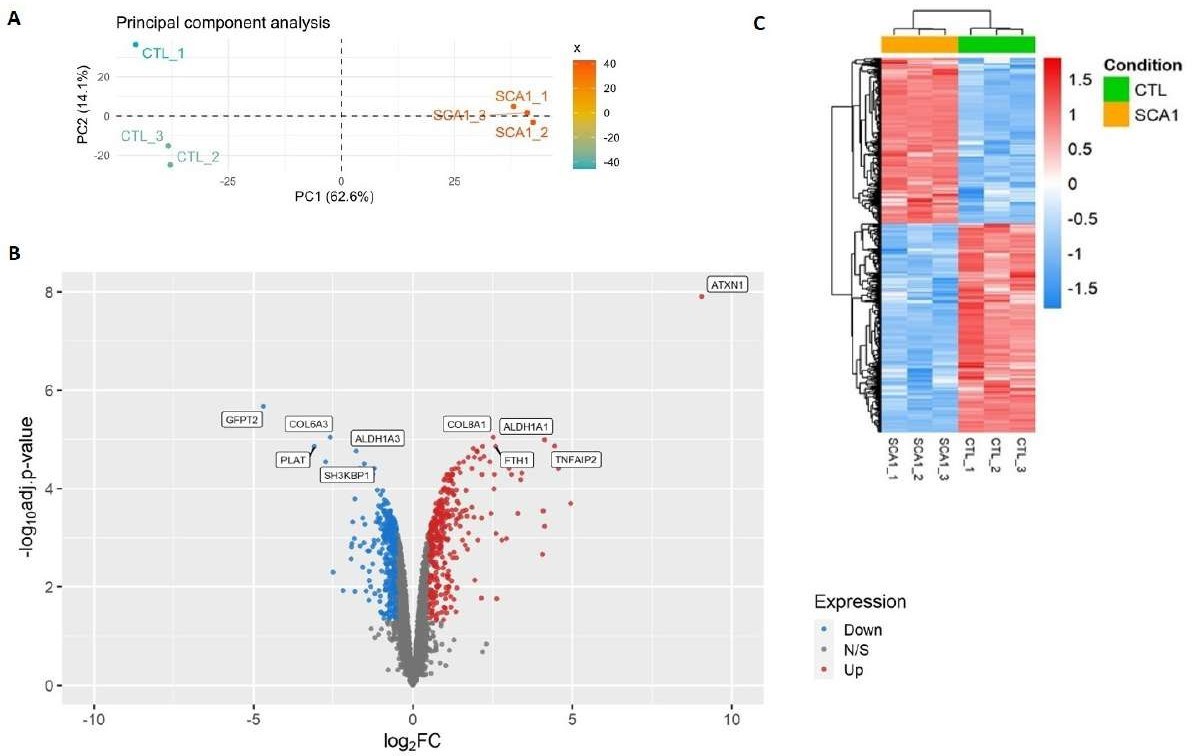

Supplement: S4 Fig — (A) SCA1 cells were efficiently discriminated from control cells (CTL) using PCA. (B) Volcano plot depicting 449 significantly downregulated proteins (blue color) and 356 significantly upregulated proteins (red color) in SCA1 cells [selection criteria (log2FC ≤ | 0.5 | , adj. p-value ≤ 0.05)]. The top 10 dysregulated proteins are highlighted in the plot. (C) Heatmap plot according to Euclidean distance indicates two distinct groups of up- and down-regulated proteins (red and blue color, respectively) in SCA1 and control cells. (JPG) [file pone.0319084.s004.jpg]

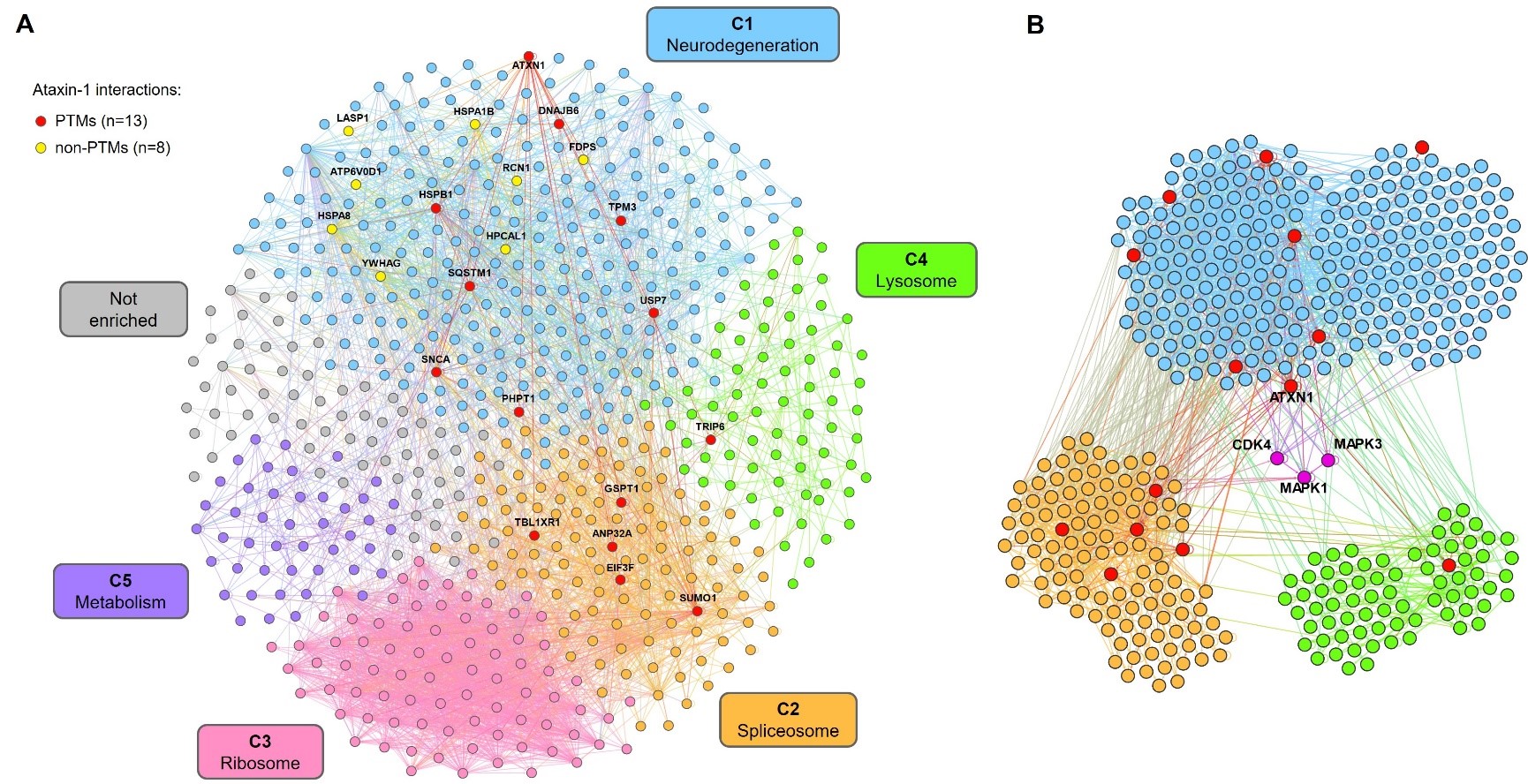

Supplement: S5 Fig — (A) PPI network of significantly dysregulated proteins in SCA1 cells. Proteins were clustered into dense communities representing functional modules. Enrichment analysis on each cluster indicated a strong association with neurodegeneration (C1), spliceosomal (C2) and lysosomal (C4) activity, ribosome assembly (C3) and metabolic pathways (C5). Ataxin-1 directly interacts with 21 proteins, 13 of which are predicted as PTM-PPIs and participate in C1, C2 and C4. Both PTM and non-PTM PPIs of ataxin-1 are highlighted with red and yellow color, respectively. (B) Identification of regulatory kinases for C1, C2 and C4 clusters, which contain the PTM-PPIs of ataxin-1. MAPK1, MAPK3 and CDK4 are significantly dysregulated in SCA1 cells. (JPG) [file pone.0319084.s005.jpg]
